# Multiparametric MRI-based radiomic model for predicting lymph node metastasis after neoadjuvant chemoradiotherapy in locally advanced rectal cancer

**DOI:** 10.1186/s13244-024-01726-4

**Published:** 2024-06-26

**Authors:** Qiurong Wei, Ling Chen, Xiaoyan Hou, Yunying Lin, Renlong Xie, Xiayu Yu, Hanliang Zhang, Zhibo Wen, Yuankui Wu, Xian Liu, Weicui Chen

**Affiliations:** 1 Department of Radiology, The Second Affiliated Hospital of Guangzhou University of Chinese Medicine, Guangzhou, China; 2 Department of Radiology, Peking University Third Hospital, Beijing, China; 3 Department of Radiology, Zhujiang Hospital, Southern Medical University, Guangzhou, China; 4 Department of Medical Imaging, Nanfang Hospital, Southern Medical University, Guangzhou, China

**Keywords:** Rectal neoplasm, Magnetic resonance imaging, Machine learning, Neoadjuvant therapy, Lymphatic metastasis

## Abstract

**Objectives:**

To construct and validate multiparametric MR-based radiomic models based on primary tumors for predicting lymph node metastasis (LNM) following neoadjuvant chemoradiotherapy (nCRT) in locally advanced rectal cancer (LARC) patients.

**Methods:**

A total of 150 LARC patients from two independent centers were enrolled. The training cohort comprised 100 patients from center A. Fifty patients from center B were included in the external validation cohort. Radiomic features were extracted from the manually segmented volume of interests of the primary tumor before and after nCRT. Feature selection was performed using multivariate logistic regression analysis. The clinical risk factors were selected via the least absolute shrinkage and selection operator method. The radiologist’s assessment of LNM was performed. Eight models were constructed using random forest classifiers, including four single-sequence models, three combined-sequence models, and a clinical model. The models’ discriminative performance was assessed via receiver operating characteristic curve analysis quantified by the area under the curve (AUC).

**Results:**

The AUCs of the radiologist’s assessment, the clinical model, and the single-sequence models ranged from 0.556 to 0.756 in the external validation cohort. Among the single-sequence models, model_post_DWI_ exhibited superior predictive power, with an AUC of 0.756 in the external validation set. In combined-sequence models, model_pre_T2_DWI_post_ had the best diagnostic performance in predicting LNM after nCRT, with a significantly higher AUC (0.831) than those of the clinical model, model_pre_T2_DWI_, and the single-sequence models (all *p* < 0.05).

**Conclusions:**

A multiparametric model that incorporates MR radiomic features before and after nCRT is optimal for predicting LNM after nCRT in LARC.

**Critical relevance statement:**

This study enrolled 150 LARC patients from two independent centers and constructed multiparametric MR-based radiomic models based on primary tumors for predicting LNM following nCRT, which aims to guide therapeutic decisions and predict prognosis for LARC patients.

**Key Points:**

- The biological characteristics of primary tumors and metastatic LNs are similar in rectal cancer.
- Radiomics features and clinical data before and after nCRT provide complementary tumor information.
- Preoperative prediction of LN status after nCRT contributes to clinical decision-making.

**Graphical Abstract:**

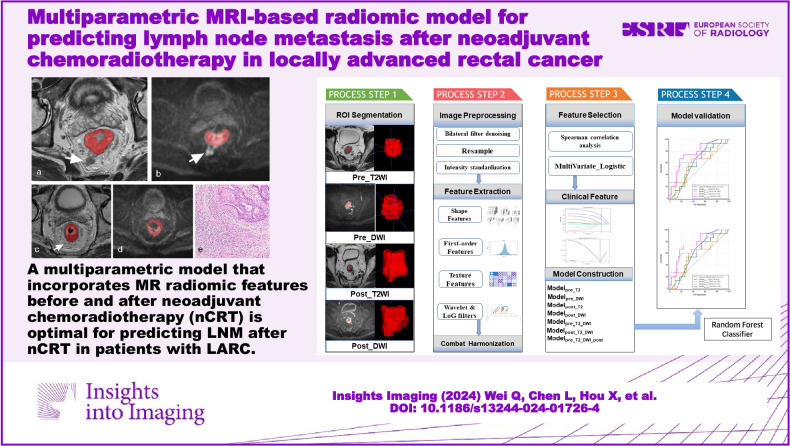

## Introduction

Locally advanced rectal cancer (LARC) refers to patients with rectal cancer (RC) with clinical (c) T3-cT4 or positive nodal status. The standard treatment strategy is neoadjuvant chemoradiotherapy (nCRT) followed by total mesorectal excision [1]. nCRT aims to achieve tumor downstaging, improve resection rate, increase sphincter preservation probability, and reduce local recurrence rate. For patients with a clinically complete response to nCRT, organ preservation strategies, such as a watchful waiting policy, could avoid radical surgery, preserve organ function, and enhance quality of life. Notably, studies suggest a possible link between the status of lymph nodes (LNs) after nCRT and the prognosis of LARC patients because complete LN regression consistently correlates with improved disease-free survival, overall survival, and reduced local recurrence and distal metastasis risk [2–4]. Chan et al found that the recurrence rate of LN-positive patients was six times higher than that of LN-negative patients; moreover, the five-year survival rate was 42% for LN-positive patients and 85% for LN-negative patients [5]. Additionally, when watchful waiting or local excision is considered, a precise assessment of LN restaging following nCRT is important. Lymph node regression after nCRT may help predict the clinical complete response of the primary tumor [6]. Conversely, LNs containing tumor cells after nCRT are a potential source of local recurrence and distant metastasis. Therefore, accurate prediction of LN metastasis (LNM) after nCRT is crucial in therapeutic decisions and for predicting prognoses for LARC patients.

At present, the preoperative evaluation of LN status and restaging following nCRT in RC relies on high-resolution magnetic resonance imaging (MRI) [7]. High-resolution T2 weighted imaging (HR-T2WI) is preferred for the evaluation of the morphological and signal characteristics of LN, such as irregular borders, uneven internal signals, and roundness. Diffusion-weighted imaging (DWI) facilitates malignant LN detection and provides biological information on cellularity. Incorporating DWI and T2WI can improve the accuracy of preoperative LNM predictions [8]. However, the reaction of LN to nCRT is heterogeneous, ranging from residual cancers to a complete fibrotic response, causing LN changes in morphology, dimension, quantity, and texture [9, 10]. In this context, visual assessment based on MRI to identify LNM following nCRT may be ambiguous, especially for small nodes (< 3 mm). Thus, there is a need for a new diagnostic method.

Radiomics extracts quantitative features from medical images and transforms them into mineable, high-dimensional data to reveal pathophysiological information about tumor heterogeneity in biomedical images [11, 12]. Studies have demonstrated that the radiomic characteristics of primary tumors can be used to predict LNM in RC [13–15]. For instance, one study reported that a radiomic nomogram based on T2WI, apparent diffusion coefficient (ADC) features, and clinical factors performed favorably [16]. Yang et al developed and validated an HR-T2WI radiomic model that could help predict the LNM of RC [17]. Furthermore, several studies focus on predicting LNM following nCRT based on multiparametric MRI using radiomics and report relatively high performances with areas under the curve (AUCs) of 0.812–0.865 in the validation cohort [18]. However, the absence of an external cohort to show how the model performs in the real world limits the clinical translation of these methods. Additionally, the radiomic models based on MR data performed before nCRT may miss key information related to LNM.

This study aimed to construct and validate multiparametric MR-based radiomic models with the pre- and/or post-nCRT information to predict LN status following nCRT in LARC patients. To obtain better predictive performance, we constructed and validated MR-based radiomics models based on various combinations obtained from pre- and/or post-nCRT information to predict LN status following nCRT in LARC patients, and compared them with the radiologist’s qualitative evaluations.

## Materials and methods

### Patients

**Fig. 1:**
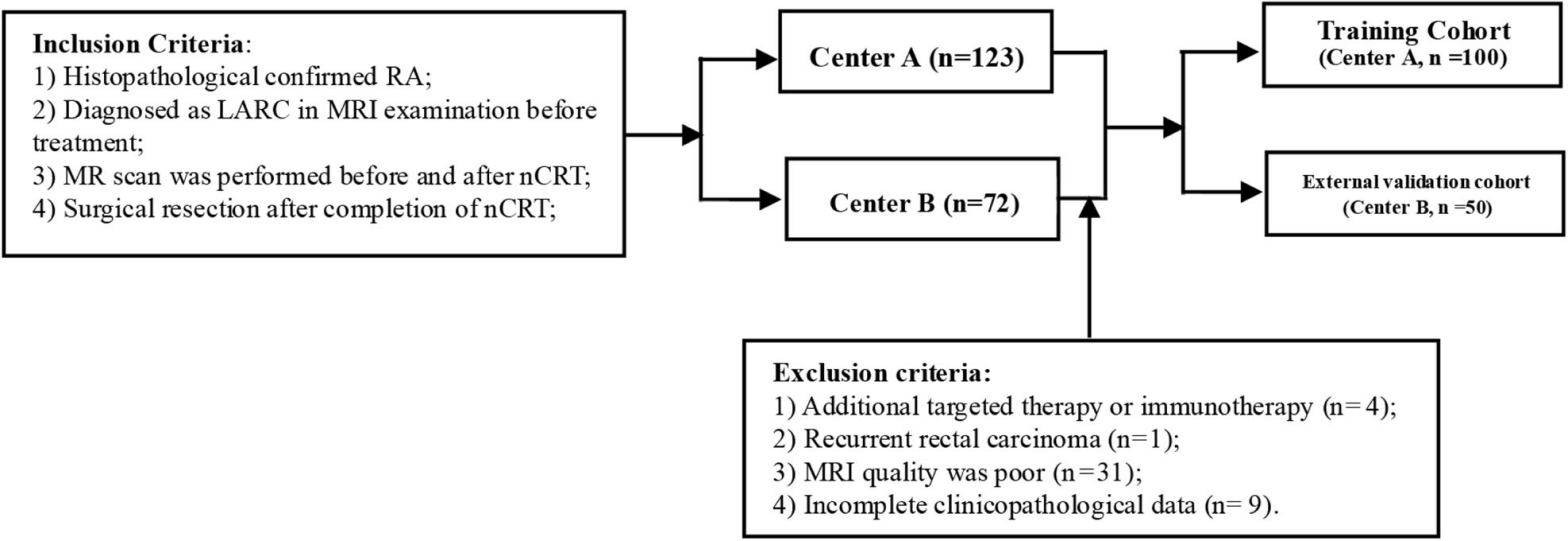
Flowchart of patient selection

The institutional review boards of the Nanfang Hospital (Guangzhou, China, center A) and the Second Affiliated Hospital of Guangzhou University of Chinese Medicine (Guangzhou, China, center B) granted ethical approval of the retrospective study and waived the need for informed consent. Between October 2017 and October 2020, consecutive LARC patients (*n* = 150) from the two medical centers were included. The inclusion criteria were: (1) histopathologically confirmed rectal adenocarcinoma; (2) diagnosed as LARC (cT3–T4 or cN1–2) at the initial treatment stages; (3) received MR scan before and after nCRT; and (4) received complete nCRT followed by surgery and confirmed by postoperative pathology. The exclusion criteria were: (1) additional targeted therapy or immunotherapy during treatment; (2) recurrent rectal carcinoma; (3) poor quality MRI; and (4) incomplete clinicopathological data. Figure 1 depicts a flowchart for patient recruitment.

The clinicopathological features of patients were obtained from their medical records. The collected data included age, gender, carcinoembryonic antigen (CEA) level before and after nCRT, tumor location, chemotherapy regimen, and tumor differentiation.

### Pathologic assessment

All patients underwent total mesorectal excision surgery after nCRT. The extent of LN dissection encompassed the following regions: perirectal LNs, internal iliac LNs, external iliac LNs, common iliac LNs, superior rectal LNs, presacral LNs, and pararectal LNs. The pathological assessment was performed on all the surgically resected LNs. The pathologic T and N stages were evaluated according to the American Joint Committee on Cancer’s Cancer Staging Manual (AJCC 8th edition) by two pathologists in consensus (X.H.D. and H.S.W., with 11 and 15 years of experience in gastrointestinal diagnosis, respectively).

### MRI acquisition and radiologist’s assessment LN after nCRT

All study participants had an MRI one week before the start of nCRT and one week before surgery; these are referred to as pre- and post-nCRT MRI, respectively. Before the MR examinations, patients received a cleansing enema but did not receive bowel preparation antispasmodic medication, or rectal distention. The imaging protocol included T2WI, DWI, and T1-weighted imaging in the oblique axial, coronal, and sagittal planes. Oblique axial and coronal sequences were angulated perpendicular and parallel to the tumor axis, respectively. Additionally, a coronal sequence parallel to the anal canal was performed in distal tumors (lower third of the rectum). Table 1 summarizes the imaging acquisition parameters for axis T2WI and DWI.

**Table 1:** MRI sequences parameters of T2WI and DWI of different MR devices

	Center A		Center B					
Manufacturer/model	Philips achieve 3.0 T		Siemens verio 3.0 T		Philips ingenia 3.0 T		Siemens prisma 3.0 T	
MR sequence	T2WI	DWI	T2WI	DWI	T2WI	DWI	T2WI	DWI
Acquisition time (ms)	04:04	01:24	03:12	01:30	02:18	01:21	03:43	01:34
DWI acquisition mode	N/A	EPI	N/A	EPI	N/A	EPI	N/A	EPI
b Values (s/mm^2^)	N/A	0, 1000	N/A	0, 1000	N/A	0, 1000	N/A	0, 1000
Repetition time (ms)	3906	2000	6350	5900	3664	3514	8040	4900
Echo time (ms)	100	60	93	83	100	81	89	53
Echo train length	21	59	28	1	17	53	19	1
Flip angle (°)	90	90	140	90	90	90	160	90
Slice thickness (mm)	3	4	3	5	3	4	3	4
Imaging frequency (Hz)	127.8	127.8	123.2	123.2	127.8	127.8	123.2	123.2
Number of average	1	2	2	2	2	2	1	1
Percent sampling (%)	100	100	80	80	84	99	80	100
(Pixel) bandwidth	218	2567	260	1628	325	2303	200	1985
Matrix	316 × 314	120 × 118	320 × 256	192 × 115	288 × 228	108 × 106	320 × 240	140 × 104
Field of view (cm)	200 × 200	240 × 240	200 × 200	270 × 360	200 × 200	320 × 320	200 × 200	180 × 320

A gastrointestinal radiologist (X.L., with 19 years of experience) retrospectively and independently reviewed MRI images to evaluate MR-based tumor regression grade (mrTRG) and LN status after nCRT, without knowing the patient’s pathological findings. mrTRG was assessed as outlined by Patel et al [19]. LN MR-restaging was assessed according to the European Society of Gastrointestinal and Abdominal Radiology (ESGAR) criterion, including size (short axis diameter > 5 mm) and nodal morphological features (shape, contour, signal intensity homogeneity on T2WI, and enhanced homogeneity) [7].

### Tumor segmentation and radiomic feature extraction

**Fig. 2:**
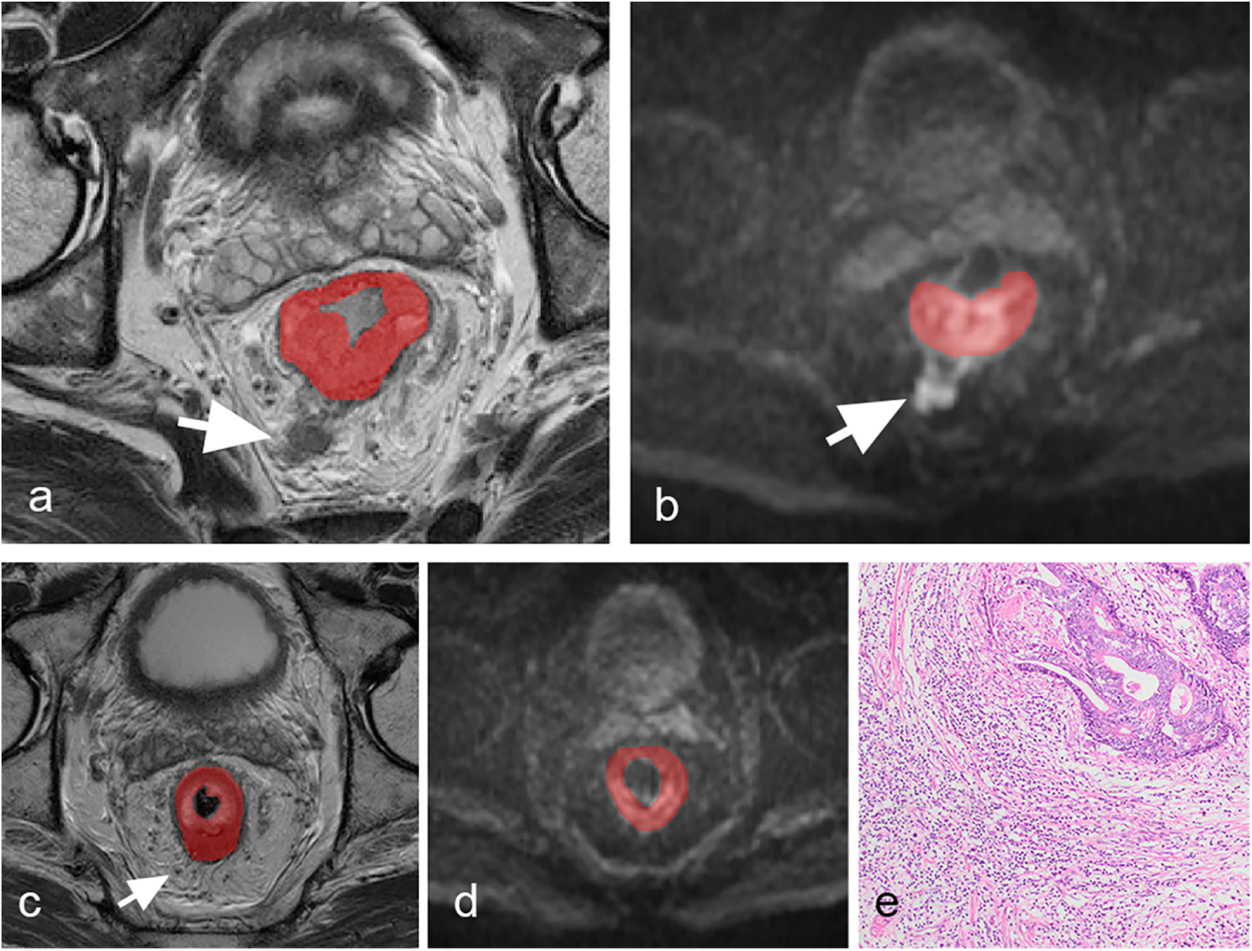
Rectal pre-and post-nCRT MRI scans in a 54-year-old man with lymph node metastasis (LNM) proven by pathology after nCRT. Regions of interest segmentation of the primary tumor on T2WI (**a**, **c**) and DWI images (**b**, **d**) before and after nCRT. Before nCRT, a suspicious metastatic lymph node (MLN) is noticed (white arrow), with high intensity in T2WI (**a**) and DWI (**b**). The suspicious MLN shrinks (< 3 mm) on T2WI (**c**) and DWI images (**d**) after nCRT. Photomicrograph (hematoxylin-eosin stain, ×100) shows the presence of residual invasive tumor cells in the LN (**e**)

The volume of interest (VOI) of the primary tumors was manually delineated in a blinded manner in the axis T2WI and DWI (*b* = 1000 s/mm^2^) before and after nCRT treatment by two radiologists (X.H. and L.C.), each with more than seven years of experience in consensus using ITK-SNAP software (version 3.8; http://www.itksnap.org/). The intestinal lumen and noninvaded rectal wall were carefully excluded from the tumor regions (Fig. 2). The radiologists were blinded to the patients’ clinicopathological information. To ensure consistency and reproducibility of extracted features, 45 patients were chosen at random to calculate the intraclass correlation coefficient (ICC); features with an ICC < 0.75 were eliminated.

Before feature extraction, the MR images were preprocessed using AK software (GE Healthcare, China) to compensate for differences owing to different protocols. The pre-processing steps were: (1) the MR images were smoothed with the bilateral filter algorithm to achieve similar noise characteristics; (2) the MR images and VOI were resampled to a uniform voxel size of 1 × 1 × 1 mm^3^ using linear interpolation and nearest neighbor interpolation, respectively; and (3) T2WI and DWI images were *Z*-score normalized to eliminate the influence of different gray value ranges.

The PyRadiomics (https://pyradiomics.readthedocs.io/en/latest/) package with the default setting was used to extract the radiomic features from the T2WI and DWI images before and after nCRT (with a fixed intensity bid width of 25). Each imaging modality yielded 960 radiomic features, for a sum of 3840 radiomic features extracted for each patient. The extracted features were: (1) 14 shape-based features; (2) 18 first-order features; (3) 68 texture features (gray-level co-occurrence matrix (GLCM), gray-level dependence matrix (GLDM), gray-level run-length matrix, and gray-level size zone matrix); (4) 688 wavelet features; and (5) 172 Gaussian Laplacian features.

### Feature selection

To standardize the radiomics features and mitigate the impact of variability among different MR scanners, *Z*-score normalization was applied to the radiomics features of each patient. Subsequently, two methodologies were explored to identify optimal features for predicting LNM in LARC patients after nCRT. Initially, pairwise matching analysis was conducted on all features, with features exhibiting a Spearman correlation coefficient > 0.70 being subjected to significant testing, where the feature with the lower *p*-value was retrained for subsequent analysis. Following this, the most predictive radiomics features were identified using multivariate logistic regression, with LNM being significantly associated with features having a *p*-value < 0.05. Moreover, given that homogeneity in the radiomics features can be influenced by center and protocol/vendor-specific dependencies, we employed the ComBat harmonization approach to eliminate batch effects arising from variations across the different centers and different MR modalities [20, 21].

### Model construction and evaluation

Random forest (RF) is a machine learning technique that uses an ensemble approach to combine decision regression trees and classification methods [22]. Compared to other classification methods, RF demonstrates enhanced efficacy in handling noisy data and outliers. Its robustness against overfitting and reduced sensitivity to input values contribute to heightened discriminative capabilities and improved precision [23, 24]. In the study, we used an RF classifier to establish machine learning models. We first constructed four single-sequence models based on T2WI or DWI features before and after nCRT (model_pre_T2_, model_pre_DWI_, model_post_T2_, and model_post_DWI_). Then, three combined radiomic models were produced by combining the features of different treatment points, including the combinations of T2WI and DWI before nCRT (model_pre_T2_DWI_), T2WI and DWI after nCRT (model_post_T2_DWI_), and T2WI and DWI before and after nCRT (model_pre_T2_DWI_post_). The clinical risk factors were selected via the least absolute shrinkage and selection operator (LASSO) method and the penalty parameters were tuned using ten-fold cross-validation. Variables with non-zero coefficients were included in the clinical model.

**Fig. 3:**
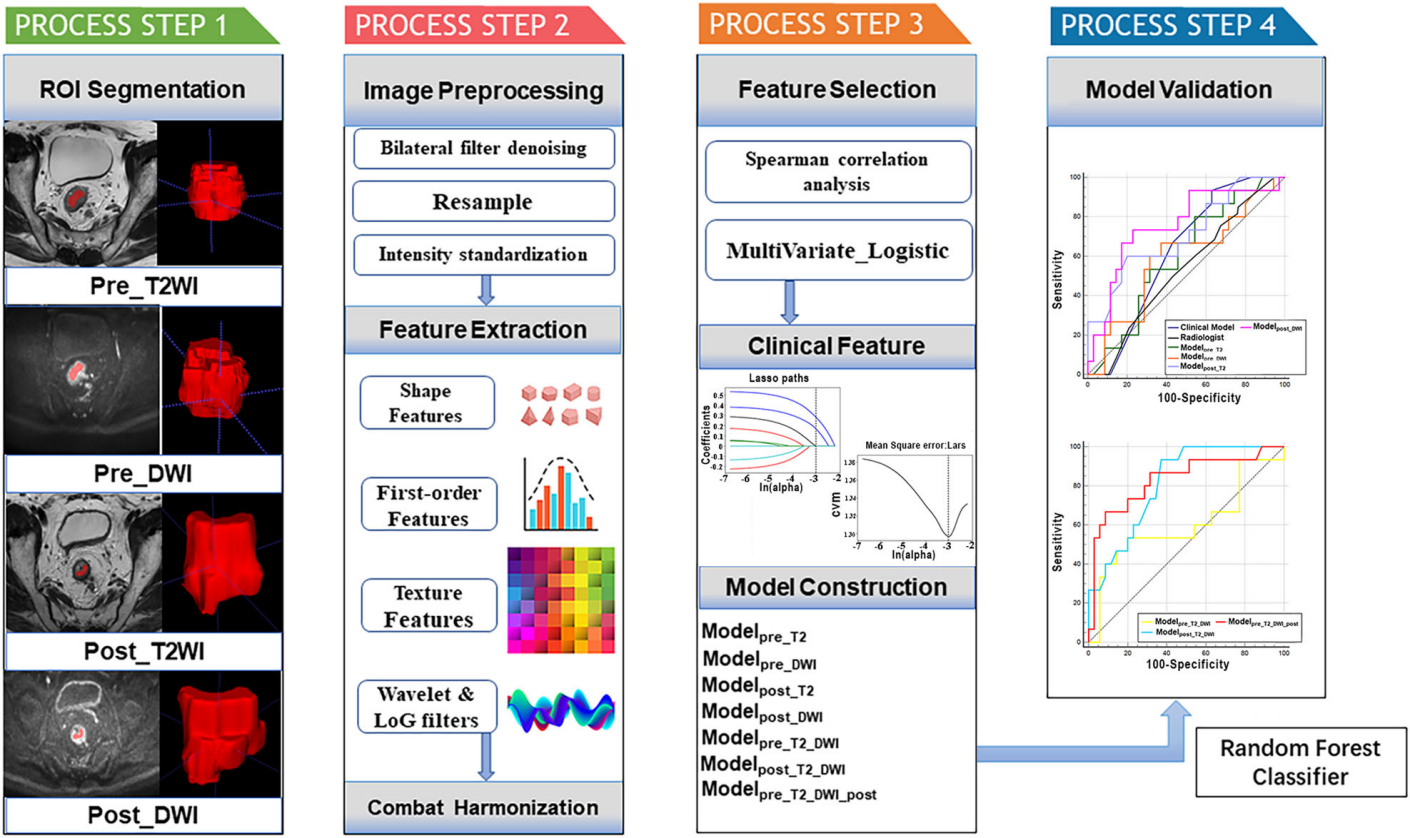
The study workflow for predicting LNM after nCRT in LARC patients

The model was trained with all the data from the training cohort and validated with data from the external validation cohort. The models’ discriminative performance was assessed via receiver operating characteristic curve (ROC) analysis, and quantified by the AUC. Figure 3 illustrates displays the pipeline for constructing and evaluating various models for predicting LNM after nCRT in LARC patients.

### Statistical analysis

SPSS (version 26.0; IBM, New York, USA) and R software (version 4.2.1; R Core Team, Vienna, Austria) were used for statistical analyses. An independent *t*-test was used to process continuous variables and the chi-square test or Fisher’s exact test was used to analyze the classified variables. DeLong’s test was utilized to evaluate differences in the predictive performance concerning the AUCs among clinical models, radiologists’ evaluations, and different radiomic models. All statistical tests were two-sided. Benjamini and Hochberg-corrected *p*-values were used to assess the feature significance for multiple comparisons.

## Results

### Patient characteristics

A total of 150 patients with LARC from two independent institutions were enrolled in this study. The training cohort comprised 100 patients from center A. Fifty patients from center B were included in the external validation cohort. Fifty-two patients with LNM status were confirmed by pathology, with a prevalence of 37% (37/100) in center A and 30% (15/50) in center B.

A significant difference was found in the CEA level after nCRT and mrTRG between the training cohort and external validation cohort (all *p* < 0.05). There were no significant differences between the training cohort and external validation cohort in gender, age, mrT Stage after nCRT, CEA level before nCRT, tumor differentiation, tumor location, or chemoradiotherapy regimen (*p* = 0.686, 0.417, 0.349, 0.236, 0.785, 0.675, and 0.669, respectively; Table 2).

**Table 2:** Clinicopathological characteristics of patients in training cohort and external validation cohort

	Training cohort (*N* = 100)			External validation cohort (*N* = 50)			
	LNM (*N* = 37)	Non-LNM (*N* = 63)	*p*-value	LNM (*N* = 15)	Non-LNM (*N* = 35)	*p*-value	*p*-value
Age (years) (mean ± SD)	54.32 ± 11.10	55.90 ± 10.24	0.472	57.73 ± 11.29	55.37 ± 11.47	0.506	0.686
Gender			0.015*			0.507	0.417
Male	24 (64.9%)	54 (85.7%)		12 (80.0%)	24 (68.6%)		
Female	13 (35.1%)	9 (14.3%)		3 (20.0%)	11 (31.4%)		
Post-nCRT mrT stage			0.742			0.043*	0.349
T1-2	3 (8.1%)	8 (12.7%)		0 (0%)	9 (25.7%)		
T3-4	34 (91.9%)	55 (77.3%)		15 (100%)	26 (74.3%)		
mrTRG			0.334			0.010*	< 0.001*
1	6 (16.2%)	18 (28.6%)		0 (0%)	8 (22.9%)		
2	17 (45.9%)	30 (47.6%)		1 (6.7%)	9 (25.7%)		
3	8 (21.6%)	10 (15.9%)		13 (86.7%)	18 (51.4%)		
4	6 (16.2%)	5 (7.9%)		1 (6.7%)	0 (0%)		
5	0 (0%)	0 (0%)		0 (0%)	0 (0%)		
Pre-nCRT CEA level			0.518			0.019*	0.236
< 5 ng/mL	14 (37.8%)	28 (44.1%)		1 (6.7%)	15 (42.9%)		
≥ 5 ng/mL	23 (62.2%)	35 (55.6%)		14 (93.3%)	20 (57.1%)		
Post-nCRT CEA level			1.000			0.021*	< 0.001*
< 5 ng/mL	35 (94.6%)	58 (92.1%)		6 (40.0%)	26 (74.3%)		
≥ 5 ng/mL	2 (5.4%)	5 (7.9%)		9 (60.0%)	9 (25.7%)		
Tumor differentiation (%)			0.099			0.360	0.785
Well differentiation	8 (21.6%)	8 (12.7%)		5 (33.3%)	5 (14.3%)		
Moderate differentiation	23 (62.2%)	51 (81.0%)		9 (60.0%)	25 (71.4%)		
Poor differentiation	6 (16.2%)	4 (6.3%)		1 (6.7%)	5 (14.3%)		
Tumor location			0.852			0.464	0.675
Low	8 (21.6%)	17 (27.0%)		5 (33.3%)	11 (31.4%)		
Middle	21 (56.8%)	34 (54.0%)		9 (60.0%)	16 (45.7%)		
Upper	8 (21.6%)	12 (19.0%)		1 (6.7%)	8 (22.9%)		
Chemotherapy regimen			0.397			1.000	0.669
Capeox	30 (81.1%)	46 (73.0%)		11 (73.3%)	24 (68.6%)		
Capecitabine	1 (2.7%)	6 (9.5%)		2 (13.3%)	4 (11.4%)		
mFOLFOX6	5 (13.5%)	6 (9.5%)		1 (6.7%)	4 (11.4%)		
5-Fluorouraciland leucovorin	1 (2.7%)	5 (7.9%)		1 (6.7%)	3 (8.6%)		

*LNM* lymph node metastasis, *nCRT* neoadjuvant chemoradiotherapy, *TRG* tumor regression grading, *p* pathological, *CEA* carcinoembryonic antigen

* *p*-value < 0.05

### Feature selection

**Fig. 4:**
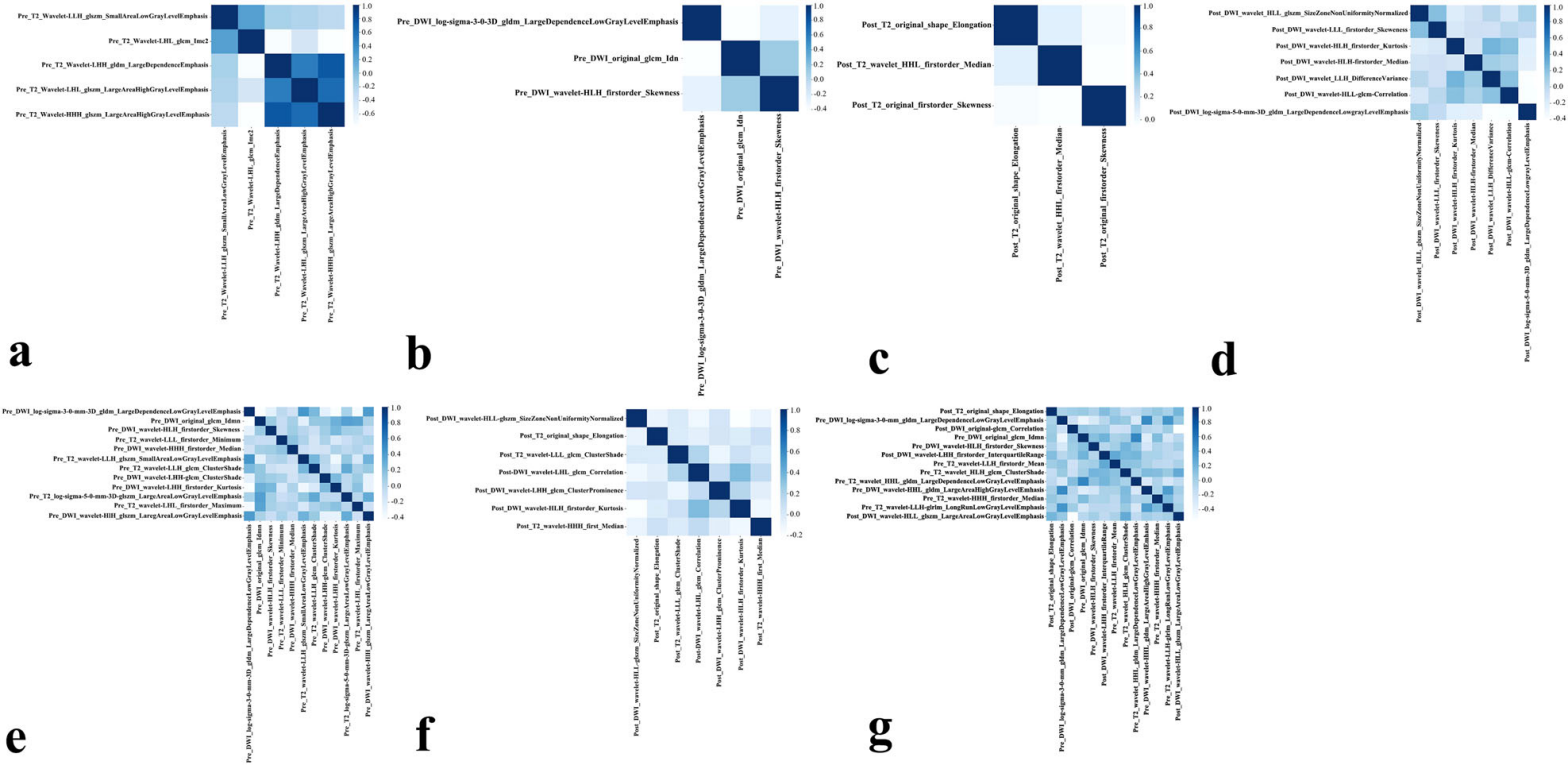
Correlation matrix plot displaying correlations between radiomic features used in different prediction models. **a** Model_pre_T2_. **b** Model_pre_DWI_. **c** Model_post_T2_. **d** Model _post_DWI_. **e** Model_pre_T2_DWI_. **f** Model_post_T2_DWI_. **g** Model_pre_T2_DWI_post_

From each MRI sequence, 960 radiomic features were extracted. Pre_T2, Pre_DWI, Post_T2, and Post_DWI were reduced to 898, 924, 864, and 955 features after excluding those with low repeatability. After the multivariate logistic regression, there were five features in Pre_T2, three features in Pre_DWI, three features in Post_T2, seven features in Post_DWI, twelve features in Pre_T2_DWI, seven features in Post_T2_DWI, and thirteen features in Pre_T2_DWI_Post. Based on the LASSO logistic regression analysis, mrTRG and gender were identified as clinical risk factors that are associated with LNM after nCRT (Fig. S1). Table 3 lists the best radiomic features of the various models and Fig. 4 depicts the correlation matrices for the selected features used in the different radiomic models.

**Table 3:** Most significant radiomics features of single-sequence models and combined models

Most significant radiomics features of single-sequence models			
Model _pre_T2_	Model _pre_DWI_	Model _post_T2_	Model _post_DWI_
wavelet-LLH_glszm_SmallAreaLowGrayLevelEmphasis	log-sigma-3-0-mm-3D_gldm_LargeDependenceLowGrayLevelEmphasis	original_shape_Elongation	wavelet-HLL_glszm_SizeZoneNonUniformityNormalized
wavelet-LHH_gldm_LargeDependenceEmphasis	original_glcm_Idn	wavelet-HHL_firstorder_Median	wavelet-LLL_firstorder_Skewness
wavelet-LHL_glszm_LargeAreaHighGrayLevelEmphasis	wavelet-HLH_firstorder_Skewness	original_firstorder_Skewness	wavelet-HLH_firstorder_Kurtosis
wavelet-HHH_glszm_LargeAreaHighGrayLevelEmphasis			wavelet-HLH_firstorder_Median
wavelet-LHL_glcm_Imc2			wavelet-LLH_glcm_DifferenceVariance
			wavelet-HLL_glcm_Correlation
			log-sigma-5-0-mm-3D_gldm_LargeDependenceLowGrayLevelEmphasis

Most significant radiomics features of combined models		
Model _pre_T2_DWI_	Model _post_T2_DWI_	Model__Pre_T2_DWI_Post_
log-sigma-3-0-mm-3D_gldm_LargeDependenceLowGrayLevelEmphasis^a^	wavelet-HLL_glszm_SizeZoneNonUniformityNormalized^c^	original_shape_Elongation^d^
original_glcm_Idmn^a^	original_shape_Elongation^d^	log-sigma-3-0-mm-3D_gldm_LargeDependenceLowGrayLevelEmphasis^a^
wavelet-HLH_firstorder_Skewness^a^	wavelet-LLL_glcm_ClusterShade^d^	original_glcm_Correlation^c^
wavelet-LLL_firstorder_Minimum^b^	wavelet-LHL_glcm_Correlation^c^	original_glcm_Idmn^a^
wavelet-HHH_firstorder_Median^a^	wavelet-LHH_glcm_ClusterProminence^c^	wavelet-HLH_firstorder_Skewness^a^
wavelet-LLH_glszm_SmallAreaLowGrayLevelEmphasis^b^	wavelet-HLH_firstorder_Kurtosis^c^	wavelet-LHH_firstorder_InterquartileRange^c^
wavelet-LLH_glcm_ClusterShade^b^	wavelet-HHL_firstorder_Median^d^	wavelet-LLH_firstorder_Mean^b^
wavelet-LHH_glcm_ClusterShade^a^		wavelet-HLH_glcm_ClusterShade^b^
wavelet-LHH_firstorder_Kurtosis^a^		wavelet-HHL_glszm_LargeAreaHighGrayLevelEmphasis^b^
log-sigma-5-0-mm-3D_glszm_LargeAreaLowGrayLevelEmphasis^b^		wavelet-HHL_gldm_LargeDependenceLowGrayLevelEmphasis^a^
wavelet-LHL_firstorder_Maximum^b^		wavelet-HHH_firstorder_Median^b^
wavelet-HLH_glszm_LargeAreaLowGrayLevelEmphasis^a^		wavelet-LLH_glrlm_LongRunLowGrayLevelEmphasis^b^
		wavelet-HLL_glszm_LargeAreaLowGrayLevelEmphasis^c^

^a^Indicates that this feature comes from DWI before nCRT.

^b^Indicates that this feature is from T2WI before nCRT.

^c^Indicates that this feature is derived from DWI after nCRT.

^d^Indicates that this feature comes from T2WI after nCRT.

### Performance of models

The assessment of LNs following nCRT by radiologist’s interpretation, based on the ESGAR criteria, yielded an AUC of 0.624 (95% CI: 0.475–0.725) in the training cohort and 0.556 (95% CI: 0.333–0.714) in the external validation cohort.

The clinical model was built based on mrTRG and gender, and the AUC of the clinical model was 0.687 (95% CI: 0.601–0.774) in the training cohort and 0.623 (95% CI: 0.503–0.742) in the external validation cohort.

For the single-sequence model, the AUC of model_pre_T2_ was 0.945 (95% CI: 0.911–0.976) in the training cohort and 0.601 (95% CI: 0.464–0.737) in the external validation cohort, respectively. The AUC generated by model_pre_DWI_ was 0.956 (95% CI: 0.925–0.983) in the training cohort and 0.589 (95% CI: 0.435–0.746) in the external validation cohort. The AUC generated by model_post_T2_ was 0.934 (95% CI: 0.891–0.970) in the training cohort and 0.710 (95% CI: 0.568–0.838) in the external validation cohort. The AUC generated by model_post_DWI_ was 0.978 (95% CI: 0.957–0.993) in the training cohort and 0.756 (95% CI: 0.616–0.877) in the external validation cohort.

**Fig. 5:**
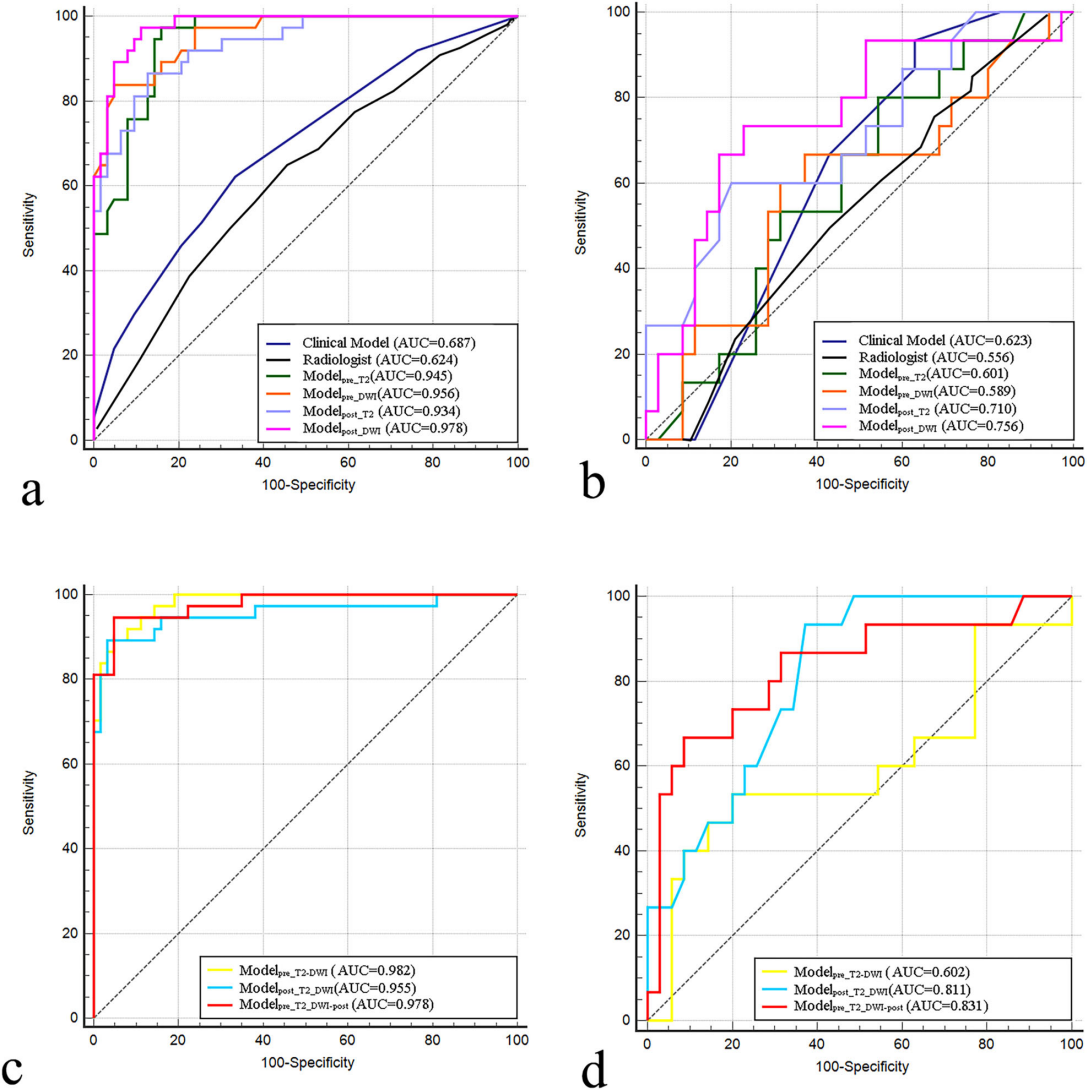
ROC curves of radiologist’s assessment and different models for predicting LNM after nCRT. Training cohort (**a**, **c**); external validation cohort (**b**, **d**)

For the combined model, the AUC of model_pre_T2_DWI_ in the training and external validation cohorts was 0.982 (95% CI: 0.964–0.995) and 0.602 (95% CI: 0.437–0.767), respectively. The AUC for model_post_T2_DWI_ in the training cohort was 0.955 (95% CI: 0.907–0.991) and 0.811 (95% CI: 0.706–0.902) in the external validation cohort. The AUC for model_pre_T2_DWI_post_ was 0.978 (95% CI: 0.956–0.996) in the training cohort and 0.831 (95% CI: 0.715–0.940) in the external validation cohort. Table 4 depicts the additional quantitative indicators for evaluating the performance of the various models. The ROC curves generated by the different models are shown in Fig. 5.

**Table 4:** Prediction performance of different models in training cohort and external validation cohort

Model	Training cohort						External validation cohort					
	AUC (95% CI)	Sensitivity	Specificity	Accuracy	NPV	PPV	AUC (95% CI)	Sensitivity	Specificity	Accuracy	NPV	PPV
Radiologist’s evaluation	0.624 (0.475, 0.725)	0.526	0.702	0.615	0.598	0.639	0.556 (0.333, 0,714)	0.526	0.610	0.578	0.564	0.578
Clinical model	0.687 (0.601, 0.774)	0.458	0.794	0.670	0.714	0.567	0.623 (0.503, 0.742)	0.667	0.571	0.600	0.800	0.400
Single-sequence models												
Model_pre_T2_	0.945 (0.911, 0.976)	0.568	0.952	0.810	0.789	0.875	0.601 (0.464, 0.737)	0.400	0.743	0.640	0.400	0.743
Model_pre_DWI_	0.956 (0.925, 0.983)	0.838	0.921	0.890	0.906	0.861	0.589 (0.435, 0.746)	0.267	0.714	0.580	0.694	0.286
Model_post_T2_	0.934 (0.891, 0.970)	0.622	0.968	0.840	0.813	0.920	0.710 (0.568, 0.838)	0.333	0.886	0.720	0.756	0.556
Model_post_DWI_	0.978 (0.957, 0.993)	0.811	0.968	0.910	0.897	0.938	0.756 (0.616, 0.877)	0.333	0.886	0.720	0.756	0.556
Combined-sequence models												
Model_pre_T2_DWI_	0.982 (0.964, 0.995)	0.811	0.984	0.920	0.899	0.968	0.602 (0.437, 0.767)	0.333	0.943	0.760	0.767	0.714
Model_post_T2_DWI_	0.955 (0.907, 0.991)	0.865	0.968	0.930	0.924	0.941	0.811 (0.706, 0.902)	0.467	0.800	0.700	0.778	0.500
Model_pre_T2_DWI_post_	0.978 (0.956, 0.996)	0.811	0.984	0.920	0.899	0.968	0.831 (0.715, 0.940)	0.600	0.943	0.840	0.846	0.818

*AUC* area under the curve, *95% CI* 95% confidence interval, *NPV* negative predictive value, *PPV* positive predictive value

### Model comparison

**Fig. 6:**
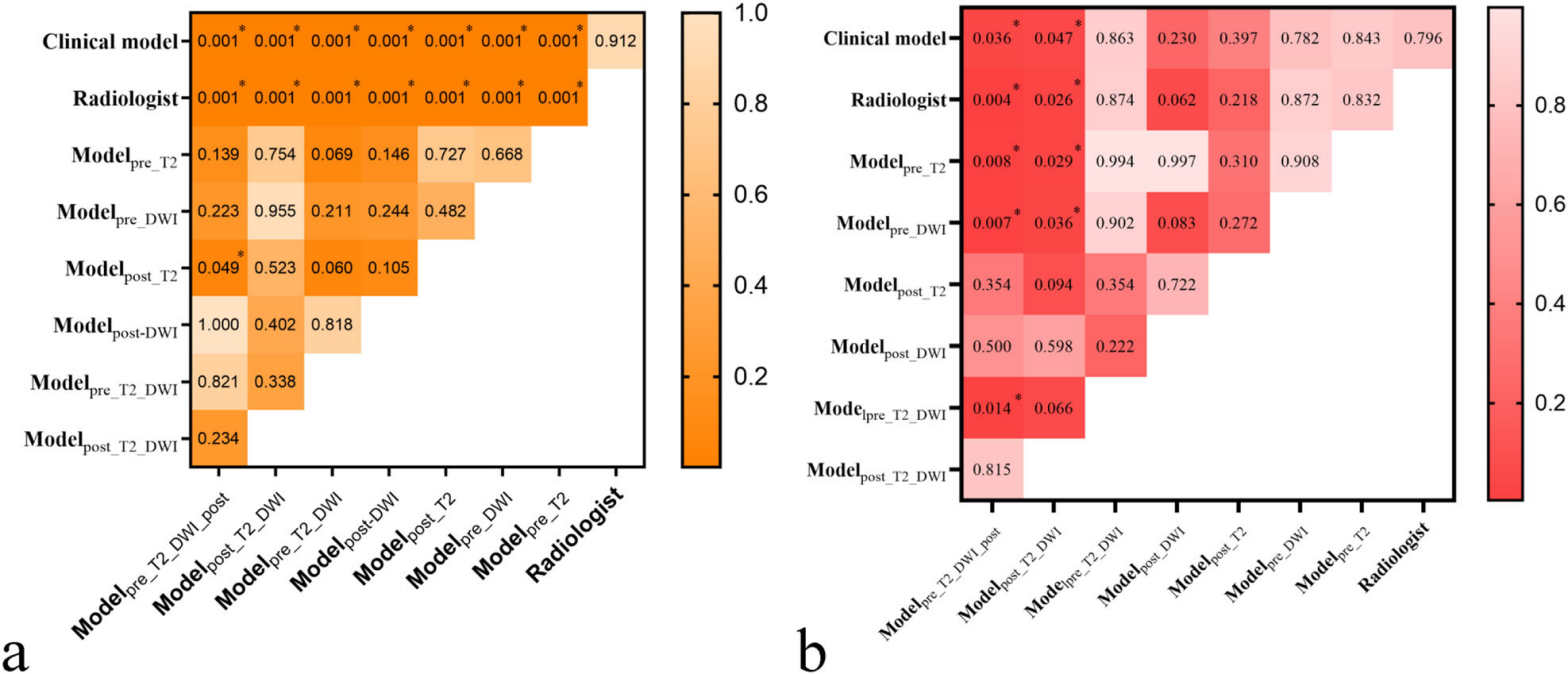
AUC comparison of radiologist’s assessment and different models with DeLong’s test in the training (**a**) and external validation cohorts (**b**)

For the training cohort, the DeLong test demonstrated that the AUCs of the radiomic models were superior to the radiologist’s assessment and clinical model (all *p* < 0.001; Fig. 6a). The AUC of model_pre_T2_DWI_post_ was better than that of model_post_T2_ (*p* = 0.049; Fig. 6a). For the external validation cohort, the AUC of model_post_T2_DWI_ was superior to the radiologist’s assessment, clinical model, model_pre_T2_, and model_pre_DWI_ (*p* = 0.026, 0.047, 0.029, and 0.036, respectively; Fig. 6b). The AUC of model_pre_T2_DWI_post_ was superior to that of radiologist’s assessment, the clinical model, model_pre_T2_, model_pre_DWI_, and model_pre_T2_DWI_ (*p* = 0.004, 0.036, 0.008, 0.007, and 0.014, respectively; Fig. 6b). The supplementary material provides a detailed comparison of various models in the training and external validation cohorts.

## Discussion

In this study, data from two centers were used to build one radiologist’s assessment model, one clinical model, four single-sequence radiomic models, and three combined-sequence radiomic models based on primary tumors to identify LNM after preoperative nCRT in LARC. An independent test set was used to assess predictive performance. To avoid confusion between pathology-confirmed metastatic LNs and MR-detected LNs, we extracted radiomic features from the primary tumor instead of individually delineating LNs. Our findings are consistent with data from previous studies that reported that the biological characteristics of primary tumors and metastatic LNs are similar [25, 26]. Our results demonstrate that the multiparametric model incorporating MR features before and after nCRT (model_pre_T2_DWI_post_) had the best diagnostic performance for predicting LNM after nCRT in the external set, with a significantly higher AUC (0.831) than those of radiologist’s assessment, clinical model, model_pre_T2_DWI_, and the single-sequence models (all *p* < 0.05). These data indicate that certain MR-based radiomic models have the potential to guide therapies for LARC patients.

Most previous radiomic studies focused on imaging data before nCRT to detect LNM [13–15]. However, tumor heterogeneity changes dynamically during treatment, and thus extracting features from imaging data before or after nCRT may miss important information about tumor changes during treatment [27]. We used both pre- and post-treatment imaging data, including T2WI and DWI, to investigate the role of radiomic features in detecting LNM. To the best of our knowledge, this is the first study to use preoperative MR data of primary tumors at two-time points to predict LNM in patients with LARC. Our findings reveal that the radiomic analysis of the baseline and follow-up MR data obtained more significant features and information on treatment-induced tumor changes. Additionally, the features of each MR sequence can still be found in the radiomic signature constructed by the Pre_T2_DWI_ Post after feature selection and eliminating redundant features, which highlights the significance of MRI parameters of both before and after nCRT in predicting LNM.

Four features were selected from T2WI and three from DWI before nCRT. After nCRT, three features were chosen from T2WI and seven from DWI. The selected features included shape, GLCM, GLDM, and wavelet features. Shape features describe the geometry of the VOI and indicate the degree of tumor complexity. GLCM and GLDM are texture-based radiomic features that characterize the intensity relationships between pairs of neighbor voxels in all spatial directions and intensity differences between neighbors, respectively [28]. Wavelet transformation offers a comprehensive spatial and frequency analysis of low- and high-frequency signals in tumor regions [29]. Tumors are biologically heterogeneous, with differences in cells, microenvironmental factors metabolism, vasculature, structure, and functions. These radiomic features reveal tumor heterogeneity at different scales, provide insights into the tumor microenvironment, and are valuable for predicting treatment responses in various tumors (non-small cell lung cancer, breast cancer, cervical cancer, and LARC).

To explore the impact of multiparametric MR data and time points on prediction accuracy, we constructed seven radiomic models, including four single and three combined-sequence models. Among the single-sequence models, model_post_DWI_ exhibited superior predictive power, with an AUC of 0.756 in the external validation set. Our results suggest that radiomic features derived from DWI might be useful for predicting LN status in LARC patients. This observation is partially consistent with data from a previous study, which reported that texture features extracted from DWI images and ADC maps can predict pathological N stages in RC, with an AUC of 0.802 [30]. DWI is a functional technique that assesses water molecule diffusion in biological tissue. The usefulness of DWI in discriminating benign from malignant tumors has been demonstrated widely. Furthermore, there is growing evidence that DWI allows for qualitative and tumor microenvironment-based quantitative assessment of the post-treatment tumor bed [31]. The histopathological characteristics of primary tumors are closely related to LNM in RC [32]. Therefore, this might be why model_post_DWI_ could successfully identify the LNM after nCRT. In combined-sequence models, the model that used T2WI and DWI features before and after nCRT had better performance than model_pre_T2_DWI_ and the single-sequence models and had high accuracy and specificity in both the training and external validation cohorts. The multi-sequence radiomic model could accurately determine LN status after nCRT, even in the absence of surgery-related clinical data. This might be interpreted that radiomic analysis based on the baseline and follow-up, therefore, may provide more significant features and information about changes resulting from treatment.

Despite the accuracy of LN restaging MRI following nCRT being better than that of baseline staging, challenges such as size overlap between malignant and reactive LNs, and fibrosis, edema, or inflammatory changes resembling tumors remain. In our study, the accuracy of the radiologist’s assessment of LN involvement was 0.578 in the external validation cohort. When constructing the clinical model, mrTRG and gender were identified as factors associated with LNM after nCRT, consistent with previous research findings [33, 34]. Newton et al developed a nomogram based on clinicopathological variables to predict LNM after nCRT in patients with LARC with a c-index of 0.71 [35], which is in line with our clinical model (AUC = 0.687). Nevertheless, the predictive performance of the clinical model is still significantly weaker than model_pre_T2_DWI_ post_ (*p* = 0.036). This might be due to clinicopathological features reflecting the coarse features of tumors, which inevitably involve clinicians’ subjective judgments of patients. In contrast, radiomic features contain multidimensional quantitative information that can more objectively and accurately reflect tumor heterogeneity and biological characteristics.

Our study has several limitations. Firstly, the sample size was small, which may affect the generalizability of the findings. Secondly, due to the retrospective nature of our study, the potential for selection bias remains, and we were unable to achieve precise alignment between pathologically confirmed LNs and those detected on MRI scans. Thirdly, we obtained data from different scanners at different centers. Although we used data pre-processing techniques such as resampling and normalization, as well as the ComBat method to eliminate batch effects, the heterogeneity of MRI scans from different centers is unavoidable. Lastly, the manual delineation of primary tumors was a time-consuming and labor-intensive process. Future studies should explore the application of deep learning for automatic VOI segmentation of RC.

In conclusion, our findings suggest that a multiparametric model that incorporates MR radiomic features before and after nCRT is optimal for predicting LNM after nCRT in patients with LARC. The model may help guide therapies and predict prognoses for LARC patients.

### Supplementary information

ELECTRONIC SUPPLEMENTARY MATERIAL

## Data Availability

The data that support the findings of this study are available on request from the corresponding author. The data are not publicly available due to privacy or ethical restrictions.

## Abbreviations

ADC: Apparent diffusion coefficient
AUC: Area under the curve
CEA: Carcinoembryonic antigen
DWI: Diffusion-weighted imaging
ESGAR: European Society of Gastrointestinal and Abdominal Radiology
GLCM: Gray-level co-occurrence matrix
GLDM: Gray-level dependence matrix
HR: High-resolution
ICC: Intraclass correlation coefficient
LARC: Locally advanced rectal cancer
LASSO: Least absolute shrinkage and selection operator
LN: Lymph node
LNM: Lymph node metastasis
MRI: Magnetic resonance imaging
mrTRG: MR-based tumor regression grade
nCRT: Neoadjuvant chemoradiotherapy
RC: Rectal cancer
RF: Random forest
ROC: Receiver operating characteristic curve
T2WI: T2-weighted imaging
VOI: Volume of interest
