# Supplementary material for: Multiparametric MRI-based radiomic model for predicting lymph node metastasis after neoadjuvant chemoradiotherapy in locally advanced rectal cancer
Source: Insights Imaging. 2024 Jun 26;15:163. doi: 10.1186/s13244-024-01726-4 (PMC11208366; doi:10.1186/s13244-024-01726-4)
Supplement: Supplementary file 1 — ELECTRONIC SUPPLEMENTARY MATERIAL [file 13244_2024_1726_MOESM1_ESM.pdf]

Multiparametric MRI-based Radiomic Model for Predicting Lymph Node Metastasis after Neoadjuvant Chemoradiotherapy in Locally Advanced Rectal Cancer

ELECTRONIC SUPPLEMENTARY MATERIAL

Supplementary Material Table S1 Pairwise Comparison of ROC Curves of Training Cohort and External Validation Cohort.

Pairwise comparison of ROC curves	Training cohort		External validation cohort	
	Z	*p*-value	Z	*p*-value
Radiologist's assessment vs Clinical model	0.186	0.912	0.268	0.796
Radiologist's assessment vs Model_{pre_T2}	4.346	<0.001*	0.197	0.832
Radiologist's assessment vs Model_{pre_DWI}	4.628	<0.001*	0.208	0.872
Radiologist's assessment vs Model_{post_T2}	5.339	<0.001*	1.218	0.218
Radiologist's assessment vs Model_{post_DWI}	5.248	<0.001*	1.677	0.062
Radiologist's assessment vs $\text{Model}_{pre\_T2\_DWI}$	5.347	<0.001*	0.125	0.874
Radiologist's assessment vs $\text{Model}_{post\_T2\_DWI}$	5.451	<0.001*	2.018	0.026*
Radiologist's assessment vs $\text{Model}_{pre\_T2\_DWI\_Post}$	5.533	<0.001*	2.178	0.004*
Clinical model vs Model_{pre_T2}	4.401	<0.001*	0.198	0.843
Clinical model vs Model_{pre_DWI}	4.934	<0.001*	0.277	0.782
Clinical model vs Model_{post_T2}	4.267	<0.001*	0.847	0.397
Clinical model vs Model_{post_DWI}	5.338	<0.001*	1.200	0.230
Clinical model vs $\text{Model}_{pre\_T2\_DWI}$	5.331	<0.001*	0.172	0.863
Clinical model vs $\text{Model}_{post\_T2\_DWI}$	4.894	<0.001*	1.984	0.047*
Clinical model vs $\text{Model}_{pre\_T2\_DWI\_Post}$	5.302	<0.001*	2.102	0.036*
Model_{pre_T2} vs Model_{pre_DWI}	0.402	0.688	0.115	0.908
Model_{pre_T2} vs Model_{post_T2}	0.349	0.727	1.015	0.310
Model_{pre_T2} vs Model_{post_DWI}	1.454	0.146	1.360	1.174
Model_{pre_T2} vs $\text{Model}_{pre\_T2\_DWI}$	1.815	0.069	0.008	0.994
Model_{pre_T2} vs $\text{Model}_{post\_T2\_DWI}$	0.313	0.754	2.190	0.029*
Model_{pre_T2} vs $\text{Model}_{pre\_T2\_DWI\_Post}$	1.481	0.139	2.673	0.008*
Model_{pre_DWI} vs Model_{post_T2}	0.703	0.482	1.098	0.272
Model_{pre_DWI} vs Model_{post_DWI}	1.164	0.244	1.733	0.083
Model_{pre_DWI} vs $\text{Model}_{pre\_T2\_DWI}$	1.251	0.211	0.124	0.902
Model_{pre_DWI} vs $\text{Model}_{post\_T2\_DWI}$	0.056	0.955	2.093	0.036*
Model_{pre_DWI} vs $\text{Model}_{pre\_T2\_DWI\_Post}$	1.219	0.223	2.709	0.007*
Model_{post_T2} vs Model_{post_DWI}	1.622	0.105	0.356	0.722
Model_{post_T2} vs $\text{Model}_{pre\_T2\_DWI}$	1.878	0.060	0.927	0.354
Model_{post_T2} vs $\text{Model}_{post\_T2\_DWI}$	0.639	0.523	1.673	0.094
Model_{post_T2} vs $\text{Model}_{pre\_T2\_DWI\_Post}$	1.973	0.049*	0.927	0.354
Model_{post_DWI} vs $\text{Model}_{pre\_T2\_DWI}$	0.230	0.818	1.222	0.222
Model_{post_DWI} vs $\text{Model}_{post\_T2\_DWI}$	0.838	0.402	0.527	0.598
Model_{post_DWI} vs $\text{Model}_{pre\_T2\_DWI\_Post}$	1.294E-14	1.000	0.674	0.500
$\text{Model}_{pre\_T2\_DWI}$ vs $\text{Model}_{post\_T2\_DWI}$	0.958	0.338	1.838	0.066
$\text{Model}_{pre\_T2\_DWI}$ vs $\text{Model}_{pre\_T2\_DWI\_Post}$	0.227	0.821	2.447	0.014*
$\text{Model}_{post\_T2\_DWI}$ vs $\text{Model}_{pre\_T2\_DWI\_Post}$	1.190	0.234	0.234	0.815

Z, Z statistic; **p*-value < 0.05.

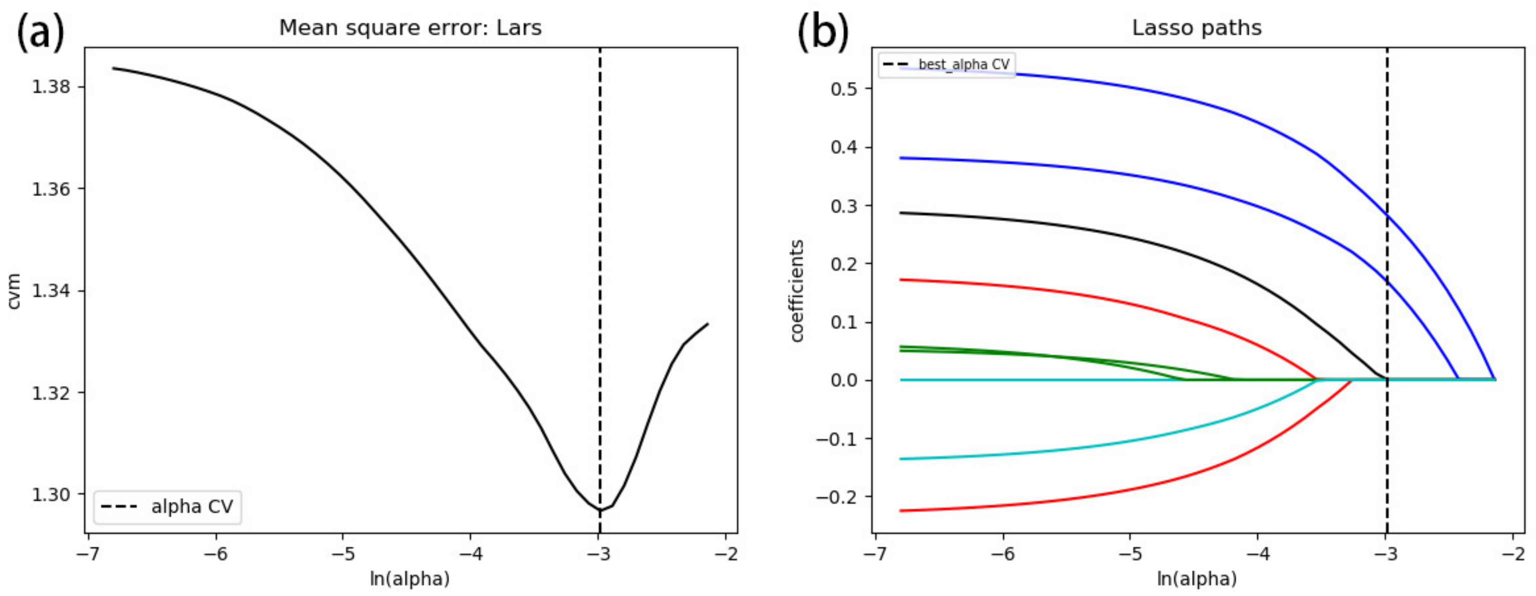

Fig S1: The clinical features were selected using the least absolute shrinkage and selection operator (LASSO) cox regression model. (**a**) Tuning parameter (λ) selection in the LASSO model used 5-fold cross-validation via minimum criteria. (**b**) LASSO coefficient profiles of the clinical features.
